# Immobilized Luminescent Bacteria for the Detection of Mycotoxins under Discrete and Flow-Through Conditions

**DOI:** 10.3390/bios9020063

**Published:** 2019-05-20

**Authors:** Olga Senko, Nikolay Stepanov, Olga Maslova, Rashid Akhundov, Anvar Ismailov, Elena Efremenko

**Affiliations:** 1 Faculty of Chemistry, Lomonosov Moscow State University, 119991 Moscow, Russia; senkoov@gmail.com (O.S.); na.stepanov@gmail.com (N.S.); olga.maslova.rabota@gmail.com (O.M.); axundovrashid@gmail.com (R.A.); anvaris@list.ru (A.I.); 2 Emanuel Institute of Biochemical Physics, Russian Academy of Science, 119334 Moscow, Russia

**Keywords:** bioluminescent bacteria, immobilized cells, mycotoxins, flow-through system, biosensitive element, analysis, hydrolysis

## Abstract

A biosensitive element in the form of bacterial *Photobacterium phosphoreum* cells immobilized in poly(vinyl alcohol) cryogel was tested for the determination of different mycotoxins under discrete and flow-through analysis conditions. The immobilized bioluminescent cells made it possible to quantify the presence of Ochratoxin A, Sterigmatocystin, Zearalenone, and Deoxynivalenon in aqueous media in a wide range of their concentrations (0.017–56 mg/L, 0.010–33 mg/L, 0.009–14 mg/L, and 0.026–177 mg/L, respectively) via measuring the quenching of cell luminescence. The flow conditions allowed the analysis sensitivity to be improved by an order of magnitude in terms of detected concentrations. Using the immobilized luminescent bacterial cells, we have shown the possibility of evaluating the efficiency of the mycotoxins’ hydrolysis under the action of enzymes. In this way, a 94 ± 4.5% efficiency of Zearalenone hydrolysis with hexahistidine-containing organophosphorus hydrolase for 1h-long treatment of the mycotoxin solution (100 mg/L) was shown.

## 1. Introduction

The problem of the contamination of various agricultural raw materials and food by mycotoxins in the modern world is becoming increasingly alarming, since, along with the development and improvement of methods for detecting mycotoxins, information is accumulated about their presence in various analyzed objects. Advances in analytical chemistry concerning the detection of mycotoxins in various environments [1], together with the latest medical and biological studies of the effect of these toxins on the human body and animals [2], indicate a high risk of these natural compounds, especially for animals that are the object of agricultural production [3,4], as well as for the persons producing and consuming agricultural products [4]. The development of modern methods of analyzing food and animal feed for the presence of mycotoxins is currently focused on creating fast and reliable control methods that are easily adaptable for various conditions [5]. Rapid screening methods are recognized in the European Union as a strategic tool for solving problems associated with mycotoxin contamination [6].

In addition, new modes of mycotoxin destruction are being actively developed [7,8,9]. In order to intensify the search for effective approaches to the destruction of mycotoxins, it is necessary to use methods for quickly assessing their efficiency, which give a prompt and reliable analytical response.

An analytical test based on the use of bioluminescent bacteria meets all of the above requirements. In particular, it is characterized by a quick response and the possibility of qualitative and quantitative analysis. Additionally, it is possible to adapt existing portable devices for its use [10,11]. An example is the commercial standardized Microtox system based on suspension bacteria *Allivibrio fischeri* (*Vibrio fischeri*) cells, which is currently used to determine the presence of a wide variety of eco-toxicants. However, other luminous bacteria have been described that can be successfully used for the same purpose [12]. Particular attention is paid to the use of biosensitive elements in the form of immobilized bioluminescent bacterial cells [13,14]. The immobilization of luminous bacterial cells makes it possible to use them under conditions in which the applicability of suspension cultures is limited, for example, in flow systems. The analysis of toxicants under flow conditions allows the minimum detectable concentrations of xenobiotics to be increased, reducing the analysis time by increasing the flow rate of the solution. In particular, this was previously shown for a biosensitive element developed on the basis of the bacterial *Photobacterium phosphoreum* cells immobilized in macroporous poly(vinyl alcohol) cryogel (PVA cryogel). It was found that such a biosensitive element can be successfully used for analyzing the toxicity of media containing heavy metal ions, phenol derivatives, and organophosphorous pesticides, both in a discrete and flow-through mode [13,14]. The PVA cryogel as a carrier positively influences the stability of the cell bioluminescent signal in the absence of toxins and does not affect the bioluminescent registration with the modern devices used in the experiments.

In general, PVA cryogel is a well-known nontoxic and chemically stable polymer matrix [15,16,17] successfully used for the immobilization of living cells of different microorganisms. It can be stored for a long time [18,19] and has been proven to be effective for various applications [20,21,22,23]. The immobilization of cells in the PVA cryogel can be achieved by the inclusion of the cells in the macroporous polymer matrix during the process of its cryoformation. The carrier matrix is formed owing to multiple intermolecular links between the polymer molecules: a lot of hydrogen bonds appear between the OH-groups of the neighboring polymer chains [24], and the cells are accumulated inside the pores of the matrix. It is known that, in their suspended form, bacterial *P. phosphoreum* cells and several other strains can be used for determining the presence of micotoxins [25]. The spectrum of mycotoxins and the range of their concentrations that can be detected using bioluminescent bacterial cells are known from various scientific publications (Table 1) [25,26,27,28,29,30,31]. However, the possibility of using bioluminescent bacterial cells in an immobilized form for the analysis of mycotoxins has not been investigated so far.

The mechanism of the influence of various toxic compounds on the bioluminescence of bacterial *Photobacterium phosphoreum* cells depends on the chemical nature of these compounds, but the sensitivity of the toxins’ determination essentially depends on the form of the cells used for the analysis (suspended or immobilized) [32]. Therefore, the purpose of this work was to assess the feasibility of using cells of bioluminescent bacteria *P. phosphoreum* immobilized in PVA cryogel for detecting several mycotoxins under discrete and flow-through conditions. In particular, it was also interesting to apply the immobilized cells of bioluminescent bacterial cells for evaluating the efficiency of mycotoxin hydrolysis under the action of enzymes. In this study, such an assessment was carried out for the hydrolysis of Zearalenone under the action of the enzyme hexahistidine organophosphorus hydrolase (His_6_-OPH).

Below, we list the novel features of this investigation compared to our previous studies on using photobacterial cells immobilized in PVA cryogel as a biosensitive element: (i) new substances such as mycotoxins were analyzed in this work. In particular, the detection of Sterigmatocystin was investigated by the bioluminescent method with photobacterial cells for the first time; (ii) the detection of mycotoxins in the flow-through regime of functioning of the biosensing apparatus was demonstrated for the first time, and the sensitivity of the detection, as well as duration of analysis, were improved under the flow conditions of analysis; and iii) the evaluation of the efficiency of mycotoxin hydrolysis as a result of enzyme action was performed using immobilized bioluminescent bacterial cells for the first time.

## 2. Materials and Methods

### 2.1. Materials

Mycotoxins (Ochratoxin A, Sterigmatocystin, Zearalenone, and Deoxynivalenon) were purchased from Sigma-Aldrich to carry out this investigation. For the experiments, concentrated solutions of mycotoxins in methanol were preliminarily prepared. Solutions of mycotoxins of the required concentration were prepared by diluting the original stock solutions of mycotoxins in methanol. In the analysis, the quenching of the bioluminescence of the immobilized luminous bacteria under the action of the methanol present in the reaction medium was taken into account.

Na_2_HPO_4_, KH_2_PO_4_ × 2H_2_O, (NH_4_)_2_HPO_4_, Pb(NO_3_)_2_, CuSO_4_ × 5H_2_O, ZnSO_4_ × 7H_2_O, glycerol, MgSO_4_ × 7H_2_O, NaCl, and HgCl_2_ were purchased from Chimmed (Moscow, Russia); peptone and yeast extract were purchased from Difco (Becton, Dickinson and Company, Franklin Lakes, NJ, USA); and poly(vinyl alcohol) 16/1 (M.w. 84 kDa) was purchased from Sinopec Corp (Beijing, China). *Photobacterium phosphoreum* B-1717 was purchased from the All-Russian Collection of Industrial Microorganisms (http://www.genetika.ru/vkpm/).

### 2.2. Culture and Growth Conditions

*P. phosphoreum* cells were grown and maintained in a submerged culture at 18 °C under agitation at 60 rpm (IRC-1-U temperature-controlled shaker, Adolf Kuhner AG Apparatebau, Switzerland). The composition of the Farghaly growth medium (g/L distilled water) was as follows: NaCl: 30.0; Na_2_HPO_4_: 5.3; KH_2_PO_4_ × 2H_2_O: 2.1; (NH_4_)_2_HPO_4_: 0.5; MgSO_4_ × 7H_2_O: 0.1; yeast extract: 1.0; peptone: 5.0; glycerol: 3.0. The cell concentration in the growing culture was determined by spectrophotometry. To convert the cell densities into the biomass concentration, the calibration curve for optical density vs. dry cell weight was used. The optical density of the culture medium was determined by spectrophotometry (Agilent UV-853 spectrophotometer, Agilent Technologies, Waldbronn, Germany) at the wavelength of 660 nm, and the cells were cultivated for 22 h to an optical density of 0.75 ± 0.05, separated from the culture medium by centrifugation (5000 rpm, 15 min., J2 21 centrifuge, Beckman, Brea, CA, USA), and afterwards used in the immobilization procedure. The procedure for immobilizing the bioluminescent cells in PVA cryogel was described previously [14]. To prepare the samples of immobilized cells, the cell biomass precipitate (with a moisture content of 78 ± 2%) obtained after centrifugation of the cell broth was thoroughly mixed with a 10% (*w*/*v*) aqueous PVA solution to obtain a 10% (*w*/*w*) concentration of bacterial cells. This mixture was pipetted into 96-well microplates (0.2 mL/well), which were placed in a freezer at −20 °C for 24 h and then thawed at +4 °C. The cylinder granules of PVA cryogel (d = 6.6 ± 0.1 mm, h = 4.9 ± 0.1 mm) formed in this way contained cells immobilized by inclusion. The average wet weight of one granule was 0.17 ± 0.001 g.

### 2.3. Luminescence Measurements

Bioluminescence of immobilized bacteria was analyzed using both a 1250 LKB-Wallac luminometer (LKB Wallac, Turku, Finland) and 3560 microluminometer (New Horizons Diagnostics Co, Columbia, MD, USA). Bioluminescence detection was performed in aqueous media based on a 2% NaCl solution at 10 ± 1 °C. The maximum level of luminescence (I_0_) was determined for 10 s at 10 °C after thermal equilibration of the flow-through system. The tests were performed in triplicate. For practical purposes, the residual intensity of bioluminescence was used (I/I_0_), which was expressed as a percentage of the baseline signal (I_0_). The residual intensity of bioluminescence (I/I_0_) was analyzed in a discrete test after the exposure of the cells to a certain mycotoxin for 0.5 h after its addition to medium containing the biosensitive element.

With the requirements of the present-day quick monitoring in mind, we assembled a biosensing apparatus to detect the mycotoxins in aqueous media in a flow-through regime. Photobacteria immobilized in PVA cryogel were used as a sensitive element in this biosensing apparatus, which was described in detail previously [13,14]. The principal setup of the biosensing apparatus for detecting mycotoxins in flow-through aqueous systems using immobilized photobacterial was as follows. To evaluate the presence of a certain mycotoxin, the sample of luminescent cells immobilized in PVA cryogel (1 granule) was placed into the analytical cell (flow cuvette of a 1250 LKB-Wallac luminometer (Turku, Finland) filled with 2% NaCl solution and localized in the thermostatted chamber of the device. Previously thermostatted (+10 ± 1 °C) sample solution containing the mycotoxin to be analyzed was pumped from a separate container with a velocity of 90 mL/h controlled by a peristaltic recycling pump (Masterflex Easy-Load II L/S, model 77200-50, Cole Parmer, Barrington, IL, USA) through the analytical cell (2 mL) for a certain time (10 min) to ensure contact of the solution with the immobilized photobacteria, and the effluent was collected in a trash container. The bioluminescent signal was permanently registered with a computer system. The data obtained (the initial and the residual levels of bioluminescence) were used to construct a graph the concentration dependencies of the luminescent cells’ characteristics for various mycotoxins. Thus, the range of detectable concentrations of mycotoxins was analyzed under the following conditions: flow medium—2% NaCl; flow rate—90 mL/h; analysis duration—10 min; temperature—+10 ± 1 °C.

### 2.4. Hydrolysis of Zearalenone Under the Action of the Enzyme His_6_-OPH

For the experiment, a 100 mg/L solution of Zearalenone in methanol and a solution of the His_6_-OPH (0.1 mg/mL) in 0.1 M phosphate buffer (pH 7.4) with an activity of 200 U/mL were used at room temperature. The procedure for enzyme production and purification was detailed in a previous paper [33]. The activity of His_6_-OPH was determined as described previously [34], with 7.8 mM aqueous Paraoxon stock solution at 405 nm using the Agilent 8453 UV-visible spectroscopy system (Agilent Technology, Waldbronn, Germany) equipped with a thermostatted analytical cell. The initial concentration of Zearalenone in the reaction medium was 65 ± 3 mg/L. The initial toxicity of this solution was evaluated under the conditions indicated above. The treatment of the mycotoxin was carried out for 1 h at room temperature without agitation, and the residual toxicity of the obtained solution was verified using immobilized luminescent cells in a discrete mode of analysis.

### 2.5. Evaluation of Zearalenone Hydrolysis by the Enzyme-Linked Immunosorbent Assay (ELISA) Test Kit

Analyses were carried out using the MaxSignal^®^ Zearalenone ELISA Test Kit (Bioo Scientific Corp, Austin, TX, USA) with a sensitivity of 0.3 ng/mL. Samples were prepared according to the instructions provided by the manufacturers of the ELISA kits. The optical density was measured at 450 nm using the microplate reader iMark (Bio-Rad Laboratories, Inc., Hercules, CA, USA) at room temperature. All the measurements were repeated three times, and the results were analyzed with the Microplate Manager^®^ 6, version 6.3.

## 3. Results

### 3.1. The Use of a Biosensitive Element for the Determination of Mycotoxins in Aqueous Solutions

**Figure 1:**
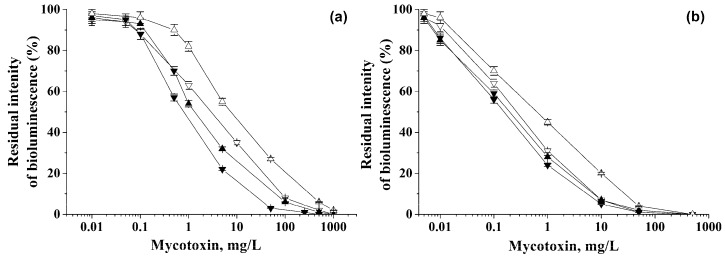
Residual intensity of bioluminescence of immobilized cells in the presence of various mycotoxins: (∇) Ochratoxin A, (▲) Deoxynivalenol, (▼) Sterigmatocystin, and (∆) Zearalenone. Analysis was carried out under discrete (**a**) and flow-through (**b**) conditions.

The linear ranges of detection of various mycotoxins were determined using *P. phosphoreum* cells immobilized in PVA cryogel (Figure 1). The dependences shown in Figure 1 and the equations describing them (Table 2) can be considered as gauge graphs.

As a result of the experiments, the lower limits of mycotoxin detection were established (Table 2), and the linear ranges were found for determining the mycotoxin concentrations using *P. phosphoreum* cells immobilized in PVA cryogel (Figure 1, Table 2).

For the flow mode, the sensitivity of mycotoxin detection turned out to be higher by a factor of 8.2–25 compared to the discrete variant. Therefore, much lower concentrations of all the tested mycotoxins were found to be detectable in the flow regime of analysis.

As it follows from the data obtained, immobilized cells of *P. phosphoreum* in discrete and flow-through modes of analysis make it possible to accurately determine the presence of mycotoxins in solutions in a wide range of concentrations with a high sensitivity.

At the same time, the detection ranges of mycotoxin concentrations under flow conditions by the use of *P. phosphoreum* cells immobilized in PVA cryogel were 1.5 orders of magnitude wider than the similar ranges established for the same immobilized cells during the discrete analysis. Additionally, these ranges were shifted towards lower concentrations.

### 3.2. Biosensitive Element in Assessment of Toxicity of the Reaction Medium Obtained after Hydrolysis of Zearalenone by His_6_-OPH

In this work, *P. phosphoreum* cells immobilized in PVA cryogel were used to evaluate the concentration of Zearalenone in the reaction medium before and after its hydrolytic treatment with His_6_-OPH, which has lactonase activity [23,24]. As a control method for the determination of Zearalenone, an ELISA method was used. It has been confirmed that the His_6_-OPH enzyme effectively degrades Zearalenone. After 1 h, the enzymatic destruction degree of this mycotoxin in the presence of His_6_-OPH was 94 ± 4.5%, and the concentration of this mycotoxin decreased from 65 ± 3 mg/L to 3.9 ± 0.2 mg/L. The nonspecific chemical hydrolysis was accounted for when the obtained result was analyzed.

The results of assessing the concentration of Zearalenone using *P. phosphoreum* bacterial cells immobilized in PVA cryogel were in very good agreement with the data obtained using the ELISA method. According to the ELISA method, after 1 h, the concentration of this mycotoxin in the presence of His_6_-OPH decreased from 65 ± 3 mg/L to 4.1 ± 0.2 mg/L.

## 4. Discussion

According to the data obtained for *P. phosphoreum* cells immobilized in PVA cryogel, the immobilized photobacterial cells in the discrete mode of analysis allow concentrations of mycotoxins to be detected in a range different from that characteristic of the suspended cells (Table 1 and Table 2).

Therefore, when using the immobilized cells (Table 2) for Zearalenone determination, the lower detection limit was 14.6 times lower, and the upper limit was 13.1 times higher, than in the case of free *P. phosphoreum* cells (Table 1). A similar trend was also observed for Ochratoxin A. Therefore, the application of immobilized cells made it possible to extend the working range of bioluminescent bacteria for the analysis of mycotoxins. In this way, the effectiveness of *P. phosphoreum* cells in an immobilized form for the analysis of mycotoxins was demonstrated in this study.

In addition, for Ochratoxin A and Zearalenone, it was shown that the concentration ranges for mycotoxin detection in the case of immobilized *P. phosphoreum* cells used in this investigation were wider than both of those known for other bioluminescent bacteria, and those established for the same luminous bacteria *P. phosphoreum* when used in suspension form to detect these two mycotoxins (Table 1 and Table 2).

We consider the results obtained for using the immobilized cells for mycotoxin analysis in a flow-through regime to be of great practical importance. The detection of mycotoxins at lower concentrations with this technique than in the discrete mode can be due to the following. The standard application of bioluminescent bacterial cells for the determination of various ecotoxicants’ presence, as a rule, consists of exposure of the cells in a solution of toxicants for 0.5 h to achieve an equilibrium state [13]. The analysis in flow mode allows the analyzed solution to be forcefully injected into the matrix of the cell carrier, and thus, ensures contact with the immobilized cells for all the mycotoxin molecules present in the analyzed sample. Therefore, the sensitivity of the bioluminescent method can be improved.

The macroporous structure of the carrier matrix used makes it possible to vary the velocity of the mycotoxin solution flow through the analytical cell over a rather wide range with immobilized cells [13,14]. The choice of the PVA cryogel as the cell carrier allows flow velocities up to 180 mL/h without problems (i.e., changing the characteristics) for the cells and polymer matrix itself. In this investigation, we used the velocity 90 mL/h; however, the applied biosensitive element can be potentially used for the analysis of toxicants at much higher flow rates (up to 180 mL/h) [13]. This is a fundamental difference between the biosensitive element used in this work and the known analytical systems based on suspension cells of bioluminescent bacteria. Therefore, it is possible to increase the rate of flow and thereby essentially reduce the time necessary for the analysis.

For practical use, it is important that the immobilized cells allow the quick detection of ecotoxicants present in the flow-through aqueous system in minimal concentrations. To quantify the applicability range of this technique, lower detection limits were determined for various mycotoxins, and calibration equations are given in Table 2. If it is known that only one mycotoxin is present in the sample, then it is possible to accurately determine its concentration.

It is known that His_6_-OPH can hydrolyze organophosphorus pesticides and mycotoxins [34,35,36,37,38]. The use of His_6_-OPH, which exhibits lactonase activity with an extended substrate spectrum of action in comparison with natural lactonases [35], also guarantees the possibility of biological neutralization of various mycotoxins containing a lactone ring. Zearalenone has a lactone cycle in its structure, and can thus be considered as a potential substrate for the His_6_-OPH enzyme, which exhibits lactonase activity.

It was recently shown that immobilized *P. phosphoreum* cells can be used for the operational control of the presence of organophosphorus pesticides in the analyzed samples taken from different objects [13]. Our results show the possibility of using immobilized bioluminescent *P. phosphoreum* cells to control the content of mycotoxins and organophosphorus compounds in analytical samples before and after using the enzymes with various catalytic activities, such as His_6_-OPH. These results can be useful to researchers engaged in the development of effective methods for the decomposition of toxins of different natures and analytical systems necessary for monitoring the effectiveness of the biological agents applied to decompose these toxins.

## 5. Conclusions

As a result of the study, it was found that *P. phosphoreum* cells immobilized in PVA cryogel can be effectively used to determine the concentration of mycotoxins, such as Ochratoxin A, Deoxynivalenol, Sterigmatocystin, and Zearalenone, in aqueous solutions in both discrete and flow-through modes of analysis. Note that the detection of Sterigmatocystin using bioluminescent bacteria has not been demonstrated previously, and the results obtained in the study of this mycotoxin are absolutely new. As compared with the discrete mode, which usually takes 30 min, in the flow mode, which can only be implemented with the immobilized form of bioluminescent cells, it is possible to determine lower levels of mycotoxins within 10 min of analysis, representing a technique that is at least three times faster. The existing potential in the use of immobilized bioluminescent cells for the determination of mycotoxins has been successfully demonstrated in evaluating the enzymatic hydrolysis of Zearalenone under the action of the enzyme His_6_-OPH.

## 6. Patents

RU Patent #2394910 Luminescent biocatalyst for the determination of toxicants.

## Figures and Tables

**Table 1:** The use of suspension cells of bioluminescent bacteria for the determination of different mycotoxins in a discrete mode of analysis.

Mycotoxin	Cells [Reference]	Limits of Detection (mg/L)
Aflatoxin B1	*Aliivibrio fischeri* [16]	1–17.1
	*P. phosphoreum* [15]	3.6–25
	*V. fischeri* (Microtox) [17]	0.005–2.98
Citrinin	*P. phosphoreum* [15]	7.00–20.0
	*V. qinghaiensis sp.* [18]	5–12
Deoxynivalenol	*V. qinghaiensis sp.* [18]	5–30
Fumonisin B1	*V. qinghaiensis sp.* [18]	5–15
Fusaric acid	*V. qinghaiensis sp.* [19]	5–40
Ochratoxin A	*V. harveyi* [20]	0.0001–0.001
	*P. phosphoreum* [15]	12.5–17.0
	*V. qinghaiensis sp.* [18]	5–11
Patulin	*P. phosphoreum* [15]
	*V. qinghaiensis sp.* [18]	5–13
Penicillic acid	*P. phosphoreum* [15]	3.14–10.7
PR-toxin	*P. phosphoreum* [15]	0.9–4.2
Rubratoxin B	*P. phosphoreum* [15]	19.69–33
T-2	*P. phosphorum Sq3* [21]	12
	*V. fischeri F1* [21]	12
Zearalenone	*V. qinghaiensis sp.* [18]	5–10
	*P. phosphoreum* [15]	9.35–13.5

**Table 2:** The results of mycotoxins’ analysis in discrete and flow-through regimes carried out using immobilized cells of bioluminescent bacteria *P. phosphoreum*.

Mycotoxin	Discrete Analysis (30 min)		Flow-Through Analysis (10 min)	
	^1^ The Equation; R^2^	Working Range, mg/L	The Equation; R^2^	Working Range, mg/L
Ochratoxin A	y = −26.87x + 62.02,	0.14–56	y = −28.80x + 34.10,	0.017–4.6
	R^2^ = 0.99		R^2^ = 0.99	
Deoxynivalenol	y = −29.10x + 59.14,	0.13–33	y = −26.50x + 31.50,	0.010–4.2
	R^2^ = 0.98		R^2^ = 0.99	
Sterigmatocystin	y = −31.61x + 51.27,	0.09–14	y = −27.50x + 29.00,	0.009–3.2
	R^2^ = 0.98		R^2^ = 0.98	
Zearalenone	y = −28.63x + 79.49,	0.64–177	y = −24.93x + 45.50,	0.026–16.7
	R^2^ = 0.98		R^2^ = 0.99	

^1^ The equations describing the calibration dependencies presented in Figure 1, for the determination of mycotoxins in discrete and flow modes of analysis using immobilized cells of *P. phosphoreum.* R^2^ is the adjusted coefficient of determination.
